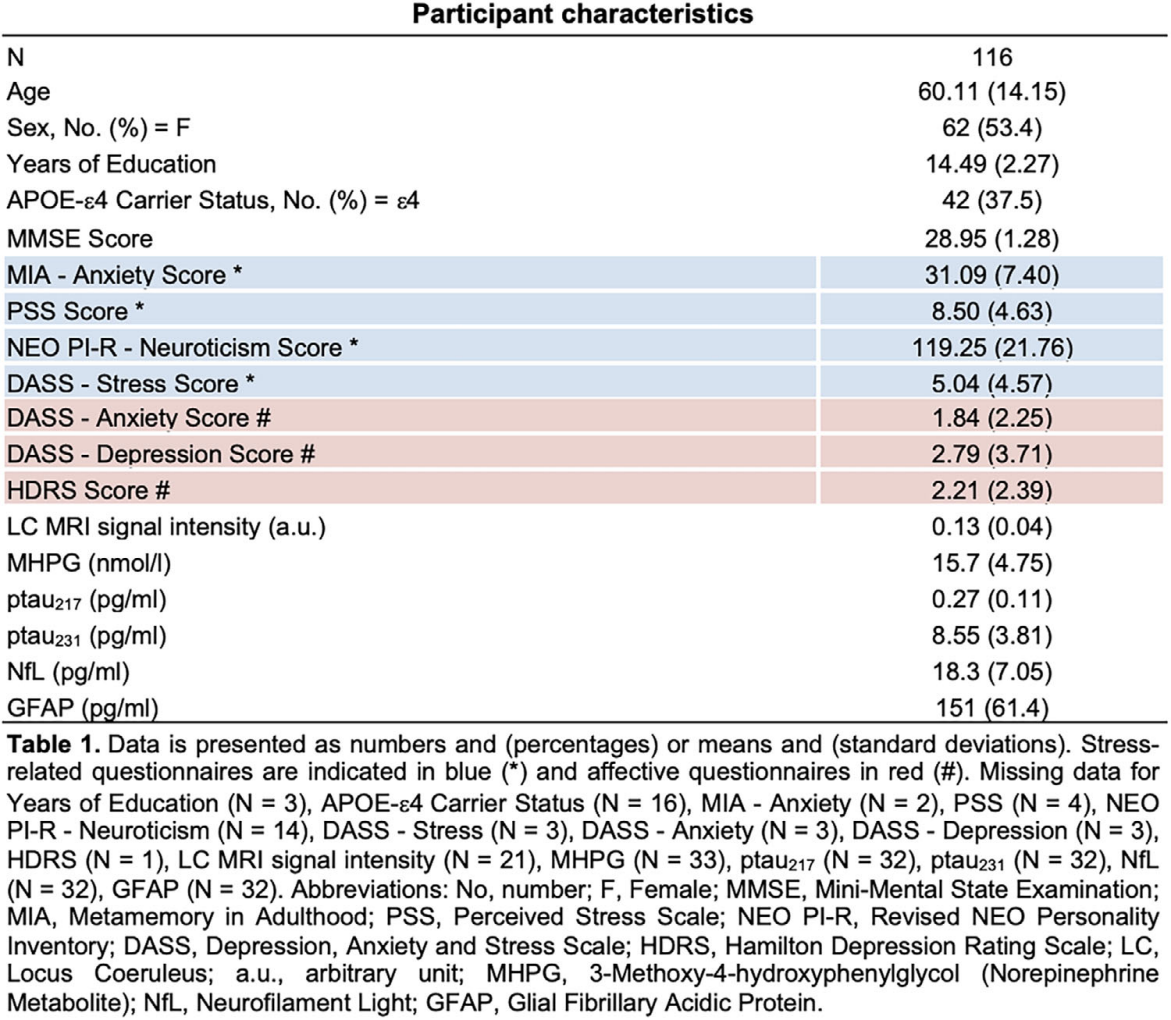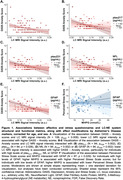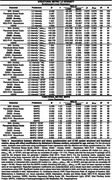# Structural and functional locus coeruleus‐norepinephrine system metrics are distinctly related to affective and stress‐related reports in the context of AD markers

**DOI:** 10.1002/alz.095603

**Published:** 2025-01-09

**Authors:** Nina Engels, Yuliya Patsyuk, Nicholas J. Ashton, Shorena Janelidze, Kaj Blennow, Oskar Hansson, Henrik Zetterberg, Joost M. Riphagen, Maxime Van Egroo, Heidi I.L. Jacobs

**Affiliations:** ^1^ Alzheimer Center Limburg, School for Mental Health and Neuroscience, Maastricht University, Maastricht Netherlands; ^2^ Athinoula A. Martinos Center for Biomedical Imaging, Massachusetts General Hospital, Charlestown, MA USA; ^3^ Department of Psychiatry and Neurochemistry, Institute of Neuroscience and Physiology, The Sahlgrenska Academy, University of Gothenburg, Mölndal, Gothenburg Sweden; ^4^ Centre for Age‐Related Medicine, Stavanger University Hospital, Stavanger Norway; ^5^ Institute of Psychiatry, Psychology and Neuroscience, Maurice Wohl Clinical Neuroscience Institute, King’s College London, London United Kingdom; ^6^ NIHR Biomedical Research Centre for Mental Health & Biomedical Research Unit for Dementia at South London & Maudsley NHS Foundation, London United Kingdom; ^7^ Clinical Memory Research Unit, Lund University, Lund Sweden; ^8^ Department of Psychiatry and Neurochemistry, Institute of Neuroscience and Physiology, The Sahlgrenska Academy, University of Gothenburg, Mölndal Sweden; ^9^ Clinical Neurochemistry Laboratory, Sahlgrenska University Hospital, Mölndal Sweden; ^10^ Memory Clinic, Skåne University Hospital, Malmö Sweden; ^11^ Clinical Memory Research Unit, Department of Clinical Sciences Malmö, Faculty of Medicine, Lund University, Lund Sweden; ^12^ Department of Neurodegenerative Disease, UCL Institute of Neurology, London United Kingdom; ^13^ Hong Kong Center for Neurodegenerative Diseases, Clear Water Bay Hong Kong; ^14^ UK Dementia Research Institute at UCL, London United Kingdom; ^15^ Harvard Medical School, Boston, MA USA; ^16^ Athinoula A. Martinos Center, Massachusetts General Hospital, Boston, MA USA

## Abstract

**Background:**

The locus coeruleus (LC)‐norepinephrine (NE) system is one of the first systems affected in Alzheimer’s Disease (AD), prior to cortical involvement. LC‐NE system dysregulation has also been associated with neuropsychiatric and stress‐related symptoms, early non‐cognitive signals of AD. This study investigates whether structural and functional LC‐NE system metrics are associated with affective and stress‐related reports among predominantly cognitively healthy adults, and whether these associations are exacerbated by AD fluid biomarkers of tau, neurodegeneration and astrocyte reactivity.

**Method:**

Cross‐sectional data from 116 life‐span study participants (cognitively healthy/MCI: N = 114/2; age range = 30‐87 years; female = 53%; Table 1) who completed affective (DASS–Stress, DASS–Anxiety, HDRS) and stress‐related (MIA–Anxiety, Perceived Stress Scale (PSS), NEO PI‐R–Neuroticism, DASS–Stress) questionnaires. Plasma 3‐methoxy‐4‐hydroxyphenylethyleneglycol (MHPG) and AD‐related markers (ptau_217_, ptau_231_, NfL, or GFAP), and 7T LC‐MRI scanning were obtained. MHPG was normalized against NE. LC MRI signal intensity was obtained using an MT‐TFL sequence, standardizing LC signal to the pontine tegmentum (reference), and creating a study‐specific LC template. Bootstrapped linear regression examined associations of affective and stress‐related questionnaires with LC MRI signal intensity alone, or interacted with AD markers. Linear regressions assessed equivalent associations with MHPG instead of LC MRI signal intensity. Analyses were based on complete cases, corrected for age and sex, and *FDR*‐adjusted.

**Results:**

Lower LC MRI signal intensity related to higher DASS–Anxiety scores (*p_FDR_* = 0.008; Table 2/Fig. 1A). This relationship was more pronounced in individuals with higher ptau_217_ (*p_FDR_* = 0.002), ptau_231_ (p = 0.028), NfL (*p_FDR_* = 0.002) or GFAP (*p_FDR_* = 0.002; Table 2/Fig. 1B‐E). Higher MHPG was linked to higher PSS scores in individuals with elevated GFAP, while higher MHPG was associated with lower PSS in the context of lower GFAP (*p* = 0.029, Table 2/Fig. 1F).

**Conclusion:**

This work suggests that structural and functional LC‐NE system metrics are distinctly related to stress and affective functioning. Lower structural LC integrity is associated with worse anxiety, particularly at elevated AD pathology levels. Among individuals with elevated astrocyte reactivity, higher LC metabolism is no longer protective against stress. These findings are consistent with previous autopsy and metabolite studies, and support the importance of the LC‐NE system in neuropsychiatric symptoms as the earliest AD manifestations.